# Preconception Micronutrient Supplementation Reduced Circulating Branched Chain Amino Acids at 12 Weeks Gestation in an Open Trial of Guatemalan Women Who Are Overweight or Obese

**DOI:** 10.3390/nu10091282

**Published:** 2018-09-11

**Authors:** Sarah J. Borengasser, Peter R. Baker, Mattie E. Kerns, Leland V. Miller, Alexandra P. Palacios, Jennifer F. Kemp, Jamie E. Westcott, Seth D. Morrison, Teri L. Hernandez, Ana Garces, Lester Figueroa, Jacob E. Friedman, K. Michael Hambidge, Nancy F. Krebs

**Affiliations:** 1 Section of Nutrition, Department of Pediatrics, School of Medicine, University of Colorado, Aurora, CO 80045, USA; MATTIE.KERNS@UCDENVER.EDU (M.E.K.); Leland.Miller@ucdenver.edu (L.V.M.); ali.palacios@yahoo.com (A.P.P.); JENNIFER.KEMP@UCDENVER.EDU (J.F.K.); Jamie.Westcott@ucdenver.edu (J.E.W.); Michael.Hambidge@ucdenver.edu (K.M.H.); Nancy.Krebs@ucdenver.edu (N.F.K.); 2 Section of Clinical Genetics and Metabolism, Department of Pediatrics, School of Medicine, University of Colorado, Aurora, CO 80045, USA; Peter.BakerII@ucdenver.edu; 3 School of Medicine, University of Oklahoma, Tulsa, OK 74135, USA; seth-morrison@ouhsc.edu; 4 Division of Endocrinology, Metabolism, & Diabetes, Department of Medicine, School of Medicine, University of Colorado, Aurora, CO 80045, USA; Teri.Hernandez@ucdenver.edu; 5 School of School of Nursing, University of Colorado, Aurora, CO 80045, USA; 6 Maternal and Infant Health Department, Institute of Nutrition of Central American and Panama, Guatemala 1188, Guatemala; agarces@incap.int (A.G.); lfigueroa@incap.int (L.F.); 7 Section of Neonatology, Department of Pediatrics, School of Medicine, University of Colorado, Aurora, CO 80045, USA; Jed.Friedman@ucdenver.edu

**Keywords:** branched chain amino acids, obesity, pregnancy, micronutrients, dried blood spot cards

## Abstract

Elevated branched chain amino acids (BCAAs: valine, leucine, and isoleucine) are well-established biomarkers of obesity-associated insulin resistance (IR). Mounting evidence suggests that low- and middle-income countries are suffering from a “double burden” of both undernutrition (growth stunting) and overnutrition (obesity) as these countries undergo a “nutrition transition”. The purpose of this study was to examine if pre-pregnancy body mass index (BMI, kg/m^2^) and a daily lipid-based micronutrient supplement (LNS, Nutriset) would lead to cross-sectional differences in circulating levels of branched chain amino acids (BCAAs) in Guatemalan women experiencing short stature during early pregnancy. Using data from an ongoing randomized controlled trial, Women First, we studied women who were normal weight (NW, BMI range for this cohort = 20.1–24.1 kg/m^2^) or overweight/obese (OW/OB, BMI range for this cohort = 25.6–31.9 kg/m^2^), and divided into two groups: those who received daily LNS ≥ 3 months prior to conception through 12 weeks gestation (+LNS), or no LNS (−LNS) (*n* = 9–10/group). BCAAs levels were obtained from dried blood spot card samples (DBS) assessed at 12 weeks gestation. DBS cards provide a stable, efficient, and reliable means of collecting, transporting, and storing blood samples in low resource or field settings. Circulating maternal leptin, adiponectin, and insulin were determined by immunoassays from serum samples collected at 12 weeks gestation. We found maternal pre-pregnancy body mass index (ppBMI) was associated with higher circulating BCAAs (*r*^2^ = 0.433, *p* = 0.002) and higher leptin/adiponectin ratio (*r* = 0.466, *p* = 0.044) in −LNS mothers at 12 weeks gestation. +LNS mothers demonstrated no correlations between BCAAs or leptin/adiponectin ratio across ppBMI suggesting LNS may be effective at improving metabolic status in OW/OB mothers during early pregnancy.

## 1. Introduction

Mounting evidence suggests that mothers and children from low- and middle-income countries are suffering from a “double burden” of both undernutrition (i.e., growth stunting or wasting) and obesity [1,2] and obesity co-morbidities [3,4] that co-exist in this population [5]. This double burden is rapidly emerging as countries undergo a “nutrition transition,” particularly in Latin America and the Caribbean as recently reviewed by Popkin and Reardon [6]. Pregnancy is a particularly sensitive time period for both the mother and developing fetus to be exposed to suboptimal nutrition [1,7,8,9,10]. The developmental origins of health and disease hypothesis has established that intrauterine exposures contribute to the pathogenesis of obesity and obesity co-morbidities in later life, and likely occurs in populations undergoing rapid transition [1]. More specifically, many retrospective and prospective studies have reported that maternal micronutrient deficiencies (hidden hunger) and caloric excess in pregnancy predict an increased risk of obesity, diabetes, and future death rate from coronary artery disease in the offspring [1,9,11,12,13]. Similarly, growth stunting in the offspring is also associated with a wide range of harmful outcomes that include increased morbidity and mortality, decreased economic status, and early onset of non-communicable diseases in later life [1,9,14,15]. 

Consequently, a “double burden” is particularly evident in populations that are challenged by both short stature and obesity, exacerbating the risk for metabolic disease and transmission to the next generation. More specifically, it has been previously reported that Guatemala has infant-stunting rates as high as 53% (length-for-age *Z*-score < −2) and has been associated with short maternal stature [14,16]. Additionally, ≈30% of infants developed obesity by 3 months of age (weight-for-length *Z*-score > 2) [14]. Moreover, maternal micronutrient deficiency, or hidden hunger, during pregnancy has been associated with increased obesity, adiposity, and metabolic disease in the offspring [1,17,18]. Conversely, maternal micronutrient supplementation has been reported to improve metabolic outcomes in animals and clinical studies [19,20,21,22]. These previous reports highlight the coexistence of under- and over-nutrition contributing to detrimental metabolic health for both mothers and infants.

Branched chain amino acids (BCAAs) are comprised of the amino acids leucine, isoleucine, and valine and are established biomarkers for obesity-associated insulin resistance in adult populations [23,24,25] and are elevated in pregnant women with obesity [26]. Measurements of BCAAs, and other amino acids, using dried blood spots (DBSs) has been employed for years as the primary means of screening newborns for metabolic disorders [27]. Only more recently have DBSs, in combination with tandem mass spectroscopy, been used for a variety of new applications [28]. DBS cards were chosen to be analyzed because they provide a stable, efficient, and reliable means of collecting, transporting, and storing blood samples [28], and may be a viable tool for low resource or field settings. Specifically, DBS collection does not require a trained phlebotomist, can be easily collected in the field at room temperature, and can be stored at room temperature for amino acid analyses as compared to serum collection and analyses. The purpose of this study was to examine if pre-pregnancy body mass index (BMI, kg/m^2^) and a daily lipid-based micronutrient supplement (LNS, Nutriset) would lead to differences in circulating levels of branched chain amino acids (BCAAs) in Guatemalan women experiencing short stature during early pregnancy using a cross-sectional study population from a larger randomized controlled trial. In the present study, LNS was administered ≥3 months prior to conception and during early gestation (+LNS) or not at all (−LNS). We hypothesized that BCAAs extracted from DBS would be elevated in OW/OB women compared with NW women, and that LNS would decrease BCAA concentrations in the OW/OB group as a sign of improved metabolic health. 

## 2. Materials and Methods

This study was a secondary analysis that was part of a large, ongoing, randomized controlled trial (RCT) called Women First (clinicaltrials.gov NCT01883193) which is investigating whether the timing of maternal nutritional intervention (lipid-based micronutrient supplement, **LNS**, Nutriset, Malaunay, France) will impact fetal linear growth [29]. The National Institute for Child Health and Human Development (NICHD) Global Network (GN) for Women’s and Children’s Research (http://gn.rti.org) was used to identify four rural sites in low resource countries to participate in the Women First study where growth stunting rates are high [29]. The sites for the primary trial are India (Belgaum, Karnataka), Pakistan (Thatta, Sind Province), Democratic Republic of the Congo (DRC, Equateur Province), and Guatemala (Chimaltenango Department). 

The present study focused on a sub-cohort of rural, Guatemalan mothers (*n* = 39) ±LNS. Women were categorized as pre-pregnancy normal weight (ppNW, BMI > 18.5–24.99 kg/m^2^) or overweight/obese (ppOW/OB, BMI ≥ 25.00 kg/m^2^) according to the World Health Organization classification. ppBMI was determined by weight and height obtained during an in-person study visit by on-site study personnel who were trained to measure subject anthropometrics using an electronic scale and adult stadiometer. Women were enrolled in the trial and they granted their informed consent for participation in accordance with the principles described in the Declaration of Helsinki. Women were then randomized to a daily lipid-based micronutrient supplementation (LNS) for ≥3 months prior to conception and the first 12 weeks of gestation (+LNS) or not at all (−LNS). Women who became pregnant prior to consuming the daily LNS for at least 3 months prior to conception were excluded from the study. Briefly, LNS is a commercially available lipid-based micronutrient supplement and has been developed for low resource settings and previously used in pregnant women in Malawi [30,31]. The nutrient composition is listed in Table S1 and has been previously published [29]. Daily LNS compliance was monitored using: (i) bi-weekly collection of empty LNS sachets performed by on-site study personnel, (ii) subjects marking off daily LNS compliance on a provided calendar, and (iii) random independent audits by other personnel. LNS compliance was determined by taking the total number of days subjects reported consuming LNS divided by the total number of days requested to consume LNS and calculated as a percentage. Subjects were matched for age, parity, pre-pregnancy (pp) height, and LNS compliance in +LNS groups (Table 1). Socioeconomic status (SES) indicator score was calculated on a score from 1–6, with 6 indicating the highest SES status. Each SES indicator equaled 1 and were tallied together to generate the SES indicator score. SES indicators included: (1) electricity, (2) improved water source, (3) sanitation, (4) man-made flooring, (5) improved cooking fuels, and (6) household assets (possessing more than one of television, telephone, bike, motorized bike or scooter, or owns a car or truck). By design, ppBMI and body weight were significantly different from each other prior to pregnancy and at 12 weeks gestation. 

### 2.1. Blood Collection and Amino Acid Profiling Using Dried Blood Spot Cards

Blood samples were collected at 12 weeks gestation by a trained phlebotomist using a venous draw (30 mL). Women were not fasted at the time of the blood draw. Approximately 0.5 mL of whole blood was applied to a Whatman 903 protein saver dried blood spot (DBS) cards (GE Healthcare Life Sciences, Pittsburgh, PA, USA) and dried for at least 4 h in a dry location. DBS cards were then stored at −20 °C and shipped on ice packs to the University of Colorado. The remaining whole blood was then immediately processed to separate serum. Samples were place on ice for 30 min to allow for clotting and centrifuged for 10 min at 2000× *g*. Serum was then transferred to cryovials and stored at −80 °C on site until shipped on dry ice to the University of Colorado and stored at −80 °C until analyzed. Serum was analyzed for total adiponectin (μg/mL, ALPCO, Salem, NH, USA), high molecular weight adiponectin (μg/mL, ALPCO, Salem, NH, USA), leptin (ng/mL, Meso Scale Discovery, Rockville, MD, USA), and insulin (μIU/mL, Meso Scale Discovery, Rockville, MD, USA) using commercially available immunoassay kits. Quality controls that were provided in each respective kit, as well as a serum pool, were used to ensure validity of each assay.

A semi-quantitative amino acid analysis was performed using DBS samples collected at 12 weeks gestation. “Semi-quantitative” is used to denote tandem mass spectometry (MS/MS) analysis in which multiple amino acids are measured simultaneously, using multiple corresponding internal standards, and quantitated relative to each internal standard. Methods and quantitative results may vary slightly from laboratory to laboratory and are not individually quantitated against a standard curve; thus, cannot be considered absolutely quantitative. However, validation of sensitivity and specificity relative to serum levels has been well established [28,32,33]. Relative differences between individuals/groups measured in the same laboratory with the same internal standards are considered biologically relevant. Briefly, two 3 mm punches were taken per subject using a 96-well plate method. Tandem mass spectroscopy electro spray ionization (MS/MS-ESI) was used to quantitate 17 amino acids. The internal standard included 8.3 micromolar of ^15^N, 2-^13^C-glycine, and 1.7 micromolar of ^2^H_4_-alanine, ^2^H_8_-valine, ^2^H_3_-leucine (co-elutes with isoleucine), ^2^H_3_-methionine, ^2^H_5_-phenylalanine, ^15^C_6_-tyrosine, ^2^H_3_-aspartate (co-elutes with asparagine), ^2^H_3_-glutamate (co-elutes with glutamine), ^2^H_2_-ornithine•2HCl, ^2^H_2_-citrulline, and ^2^H_4_, ^15^C-arginine•HCl in methanol (dilution 1:150, Cambridge Isotope Laboratories, Tewksbury, MA, USA). MS/MS-ESI was performed using API 2000 System (AB Sciex, Framingham, MA, USA), with Agilent 1100 series liquid chromatography (LC) system (Santa Clara, CA, USA), and Perkin Elmer Series 200 (Waltham, MA, USA)auto-sampler for 96-well microtiter plates. Software for analysis included Analyst v1.4.2 and ChemoView v2.0 (AB Sciex, Concord, ON, Canada). Data were analyzed for quality control using methods standard to the Children’s Hospital Colorado Biochemical Laboratory. Amino acid analysis was based on established MS/MS-ESI DBS analytic methods [32,33,34]. The lab itself is monitored and regulated by the Center for Clinical Standards and Quality through the Clinical Laboratory Improvement Amendments (CLIA). The laboratory is CLIA certified, and amino acid results from the dried blood spot methods used here are reported clinically on a routine basis.

### 2.2. Statistical Analysis

Two-way ANOVA was used to assess differences between the baseline and 12 weeks gestation subject characteristics and to test the relationship between BCAAs and ppBMI (BMI as a categorical variable) and LNS, followed by Tukey’s post hoc testing for multiple comparisons. Data are presented as means ± SEM. Pearson correlations were performed to test for associations between maternal ppBMI and serum parameters at 12 weeks gestation. The false discovery rate (FDR) for amino acid analyses was determined using the Benjamini–Hochberg procedure [35], with a significant false discovery rate (FDR) set at *p* ≤ 0.05. Linear regression analysis was used to evaluate relationships between the amino acids and maternal ppBMI. ppBMI was used as a continuous variable. To examine if LNS could alter these relationships, data from the +LNS and −LNS groups were analyzed together using unadjusted regression models that included parameters for the differences in the slopes and intercepts of the +LNS and −LNS regression lines. Residuals were examined to confirm compliance with regression assumptions. Statistical significance was set at *p* ≤ 0.05. Statistical analyses were performed using GraphPad Prism 7 (GraphPad Software, La Jolla, CA, USA) and R statistical computing software (The R Foundation, Vienna, Austria) [36]. 

## 3. Results

### 3.1. Elevated BCAAs in OW/OB Mothers and Maternal ppBMI Positively Correlated with the Leptin/Adiponectin Ratio at 12 Weeks Gestation in −LNS Groups

**Figure 1:**
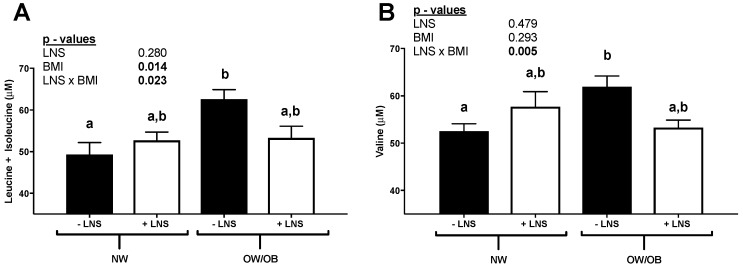
Branched chained amino acids levels (**A**) leucine + isoleucine and (**B**) valine. Data are expressed as mean ± SEM. Statistical differences for branched chain amino acid levels were determined using two-way ANOVA to examine differences due to maternal lipid-based micronutrient supplementation (LNS) and pre-pregnancy BMI category. Tukey’s post hoc testing was performed for multiple comparisons. Statistical significance was set at *p* ≤ 0.05. Different letter superscripts denote significance.

Circulating BCAAs were elevated in OW/OB not receiving LNS compared to NW as shown in Figure 1A,B. Table 2 shows serum biomarkers of maternal metabolic health. A positive correlation between maternal ppBMI and the leptin/total adiponectin ratio was found only in the −LNS group. There were multiple correlations with other amino acids that can be found in Table S2. Analysis of correlations between BCAA and serologic markers of insulin resistance and all other amino acids are shown in Table S3. BCAA (either in sum or individually) did not correlate with serum metabolic markers, but correlated positively with most of the other amino acids measured, with the exception of glutamine, arginine, and argininosuccinate, in subjects without LNS supplementation.

### 3.2. Maternal ppBMI Correlated with the Leptin/Adiponectin Ratio Was Not Correlated with BCAAs in +LNS Mothers

**Figure 2:**
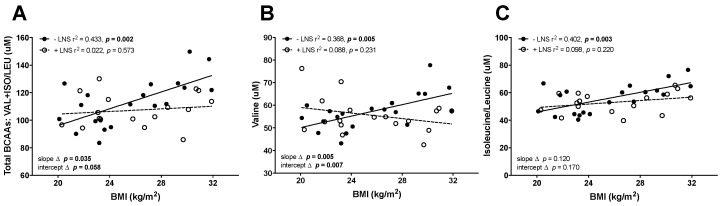
Comparative regression analysis was used to assess associations between branched chain amino acid concentrations and maternal pre-pregnancy BMI for (**A**) total branched amino acids, (**B**) valine, and (**C**) isoleucine and leucine in mothers who consumed LNS (*n* = 19 mothers) or no consumption of LNS (*n* = 20 mothers) at 12 weeks gestation. Significance was set at *p* ≤ 0.05.

Significant interactions were observed between maternal ppBMI and LNS for total BCAAs and valine alone (Figure 2). Slopes between LNS groups were significantly different from each other as shown in Figure 2A–C (*p* values ≤ 0.05), suggesting LNS may alter BCAAs. Maternal ppBMI and leptin/total adiponectin ratio were positively correlated despite LNS supplementation, while leptin was positively correlated only in the group receiving LNS (Table 2). There was no correlation between ppBMI and serum insulin. Maternal ppBMI correlations with other amino acids in subjects not receiving LNS were absent in those on supplementation (Table S2). There were several significant correlations between BCAA and maternal serum markers of metabolic health in women supplemented with LNS versus those that were not (Table S3). Mothers supplemented with LNS demonstrated negative correlations between BCAA (specifically leucine and isoleucine) and high molecular weight adiponectin, and positive correlations with leptin and the leptin/total adiponectin ratio.

## 4. Discussion

Obesity and type 2 diabetes are currently on the rise at alarming rates in low- and middle-income countries that are undergoing a nutrition transition to a Westernized diet, such as Guatemala [1,4,14]. Unfortunately, these metabolic diseases are often exacerbated by other underlying health conditions like stunted growth or a short stature. Growth stunting is associated with increased morbidity and mortality, decreased economic status, and early onset of non-communicable diseases [1,9,14,15]. The present study is the first, to our knowledge, to assess cross-sectional differences in BCAAs and other essential and nonessential amino acid levels between NW and OW/OB mothers during early pregnancy in a low- and middle-income country. We found higher maternal pre-pregnancy body mass indices (ppBMI) were associated with elevated BCAAs in un-supplemented mothers at 12 weeks gestation. This is in agreement with previous reports in OW/OB subjects, and suggestive of insulin resistance [23]. Moreover, we performed cross sectional analyses examining the differences between mothers who consumed, or did not consume, LNS on BCAA levels and other serum metabolic parameters at 12 weeks gestation. We found the ppBMI was not associated with BCAAs in the +LNS group.

Amino acid concentrations were previously assumed to be uniform and remain relatively stable during pregnancy [37,38,39,40,41]. The notion that BCAA levels may be elevated in relation to insulin resistance during pregnancy arose in the 1980s [39,40], but differences in BCAAs and other amino acid concentrations related to gestational diabetes mellitus (GDM) have been inconsistent in late pregnancy and measurements in early pregnancy are limited [42]. The earliest time in pregnancy in which amino acid profiles have been assessed is 12 weeks gestation [43]. Elevations in BCAA have also been shown in women with GDM into the third trimester [44,45] and at delivery [46]. These elevations were positively correlated with neonatal umbilical cord vein levels [46,47], neonatal weight and adiposity [44,46], and long-term offspring obesity risk in childhood [48]. Our findings are the first to suggest that higher maternal ppBMI is correlated with higher circulating levels of BCAAs as early as 12 weeks gestation, and that this elevation may be modifiable. In the present study, implications of BCAA elevations in relation to maternal ppBMI and maternal metabolic impairment were supported by other serologic markers of insulin resistance including an elevated leptin/adiponectin ratio.

BCAAs have become reliable biomarkers for obesity [49,50,51] and obesity-induced insulin resistance [23,52,53], and are considered one of the first signs of a metabolic syndrome [52,54,55,56]. Elevations in BCAA can be used to monitor glycemic control in established diabetes [57,58]. BCAAs decline with weight loss following gastric bypass surgery [59,60,61], improved metabolic health in obese individuals [58,62,63], and improved glycemic control in pregnancies of mothers with insulin dependent diabetes [64]. Positive correlations of BCAAs with ppBMI in this study, and reduced BCAAs in subjects who consumed LNS, suggest an improvement in metabolic health with LNS supplementation. This is supported by markers of improved metabolic health in the +LNS group (lower adiponectin and higher leptin and leptin/adiponectin ratio) that significantly correlated with BCAA concentrations. Leptin was typically increased, and adiponectin levels were decreased, with obesity. These differences were not seen in the un-supplemented group. Taken together, these findings suggest that an important risk factor associated with the development of insulin resistance may be modifiable by simple and affordable dietary means in low- and middle-income countries 

Another notable aspect of this study regards the utilization of DBS cards that are cost effective, can be collected in field settings, and can be easily transported and stored. In addition, a widely available and standardized assay for amino acid screening was used in the present study and can be used for both mother and infants. DBS cards are utilized throughout the United States in the screening of newborns for inborn errors of metabolism and analysis of key amino acids in pathways of energy and nutrient metabolism. While maternal BMI is a clear and measurable risk factor for maternal–infant metabolic risk, DBS analysis of established amino acid biomarkers of metabolic health offers a potentially stable and inexpensive means of assessing maternal metabolic status during pregnancy. DBS samples are stable for long periods of time at room temperature, can be easily transported and stored, and results can be generated in a high throughput fashion within minutes to hours of receiving samples. This secondary analysis study is the first to demonstrate the potential utility of DBSs in assessing metabolic health in mothers during pregnancy in a low resource setting.

There were limitations of the present study. Maternal amino acid profiles and serum parameters were measured using nonfasted samples that can impact concentrations of amino acids and serum parameters; however, all women were nonfasted. We did not have the capacity to analyze whether elevations in BCAAs correspond to results from an oral glucose tolerance test. We only examined a small sub-cohort of women from the Women First study as a secondary analysis, and likely did not have the statistical power to detect associations for some variables. Moreover, potential confounders were not controlled for in the unadjusted linear regression model which may limit the interpretability of our results. However, our study groups were well-matched for several maternal characteristics including socioeconomic status and LNS compliance, as shown in Table 1. This was an open trial where both study personnel and participants were aware of being in the treatment group; there was not a placebo supplement that would have aided in blinding the treatment. Mechanistic data are lacking to suggest how each biomarker independently may differ relative to differences in maternal BMI, insulin sensitivity, or nutritional status during pregnancy. Similarly, the LNS contained several micronutrients. Determining which component(s) may be influencing alternations in BCAAs relative to maternal ppBMI was not possible. Despite limitations, this study is unique, and the results are sufficiently promising to expand these findings to other facets of the Women First study. Additional analyses of maternal and infant samples and outcomes in the larger Women First study are currently underway and may clarify the interactive differences between maternal BMI, nutritional supplementation, and differences in serum amino acids.

## 5. Conclusions

This study addresses a population experiencing the double burden of both stunted growth and obesity and offers salient findings with regard to metabolic biomarkers in NW and OW/OB mothers with and without preconception and early gestation LNS. In particular, this study adds to the mounting literature implicating branched chain and other amino acids as markers of metabolic health in pregnancy and suggests that differences are dynamic based on maternal nutritional status prior to and during early pregnancy. Moreover, we are the first to utilize DBS samples to analyze whole blood amino acid concentrations and use a maternal preconception and early gestation LNS to test for differences in metabolic parameters at 12 weeks gestation in a low- and middle-income country. These preliminary findings may inspire future analysis of other key analytes such as vitamins, minerals, and serum markers of maternal health, to more deliberately monitor pregnancies and maternal health outcomes in low resource settings.

## Figures and Tables

**Table 1:** Maternal characteristics prior to pregnancy and 12 weeks gestation.

	BMI (<25) = NW		BMI (≥25) = OW/OB		*p*-Value		
Maternal Characteristics	−LNS (*n* = 10)	+LNS (*n* = 9)	−LNS (*n* = 10)	+LNS (*n* = 10)	LNS	BMI	LNS × BMI
**Maternal Characteristics: Pre-Pregnancy**							
**Age (years)**	23.8 ± 1.4	23.8 ± 1.6	24.7 ± 1.3	25.7 ± 1.5	0.728	0.333	0.728
**Parity**	1.6 ± 0.2	1.4 ± 0.3	1.6 ± 0.3	1.5 ± 0.3	0.605	0.863	0.863
**Ht (cm)**	146 ± 1	145 ± 2	145 ± 1	142 ± 2	0.305	0.295	0.559
**BW (kg)**	47.6 ± 1.1	47.1 ± 1.5	60.8 ± 1.6	58.6 ± 1.6	0.384	**<0.001**	0.571
**BMI (kg/m^2^)**	22.3 ± 0.4	22.3 ± 0.4	28.8 ± 0.7	28.9 ± 0.6	0.935	**<0.001**	0.892
**Compliance (%)**		77.4 ± 5.6		72.8 ± 4.7	0.533 *		
**SES**	3.6 ± 0.3	3.9 ± 0.4	4.0 ± 0.2	3.2 ± 0.5	0.466	0.679	0.125
**Maternal Characteristics: 12 Weeks Gestation**							
**Age (years)**	24.0 ± 1.4	24.4 ± 1.8	25.1 ± 1.3	25.9 ± 1.4	0.680	0.399	0.906
**BW (kg)**	47.1 ± 1.1	47.9 ± 1.6	59.8 ± 1.2	58.1 ± 1.8	0.764	**<0.001**	0.407
**BMI**	22.1 ± 0.4	22.5 ± 0.5	28.4 ± 0.6	28.7 ± 0.8	0.536	**<0.001**	0.921

Maternal anthropometric data was collected from mothers prior to pregnancy and at 12 weeks gestation by on-site study personnel. Data are shown as means ± standard error of the mean (SEM). LNS = lipid-based micronutrient supplementation, NW = normal weight, OW/OB = overweight/obese, Ht = height, BW = body weight, BMI = body mass index, and SES = socioeconomic status indicator score. Statistical differences were determined using a two-way analysis of variance for all parameters except Compliance. * Student’s *t*-test was performed to determine statistical differences for LNS Compliance. Significance was set at *p* ≤ 0.05 and is denoted in bold.

**Table 2:** Regression analysis of serum parameters in relation to maternal pre-pregnancy body mass index (ppBMI) at 12 weeks gestation was performed using Pearson correlations. “*r*-value (*p*-value)” are listed, and color coded (green = positive) for direction of correlation if the *p*-value ≤ 0.05.

			(−)LNS	(+)LNS
Category	Pathway	Variable	vs. ppBMI *r*-Value (*p*-Value)	vs. ppBMI *r*-Value (*p*-Value)
Serum	NA	**Total Adiponectin (µg/mL)**	−0.323 (0.164)	−0.312 (0.194)
Serum	NA	**HMW Adiponectin (µg/mL)**	−0.107 (0.662)	−0.39 (0.099)
Serum	NA	**%HMW/Total Ratio**	0.383 (0.095)	−0.372 (0.117)
Serum	NA	**Leptin (ng/mL)**	0.143 (0.547)	0.492 (0.033)
Serum	NA	**Insulin (µIU/mL)**	0.194 (0.413)	0.266 (0.271)
Serum	NA	**Leptin/Total Adipo Ratio**	0.466 (0.044)	0.512 (0.025)

## Supplementary Materials

The following are available online at http://www.mdpi.com/2072-6643/10/9/1282/s1, Table S1: Nutrient Content of the Lipid-based Micronutrient Supplement (20 g), Table S2: Regression analysis of leptin, adiponectin, insulin, and other amino acids in relation to maternal pre-pregnancy body mass index (ppBMI), taken at 12 weeks (12 weeks) gestation. Table S3: Correlations between branched chain amino acids ±LNS and serum parameters and other amino acids at 12 weeks gestation.
