# Supplementary material for: Preconception Micronutrient Supplementation Reduced Circulating Branched Chain Amino Acids at 12 Weeks Gestation in an Open Trial of Guatemalan Women Who Are Overweight or Obese
Source: Nutrients. 2018 Sep 11;10(9):1282. doi: 10.3390/nu10091282 (PMC6165402; doi:10.3390/nu10091282)
Supplement: Supplementary file 1 [file nutrients-10-01282-s001.docx]

Supplementary Material

Supplementary Results

Other amino acid concentrations, besides BCAAs, were captured through dried blood spot MS/MS analysis, and were positively correlated with maternal ppBMI in subjects without LNS supplementation. In Supplemental Table 2, maternal ppBMI was positively correlated with tyrosine, glutamate, alanine, methionine, serine, ornithine, and citrulline. All of these correlations were not significantly correlated with maternal ppBMI in the supplemented group. Arginosuccinic acid was the only amino acid that was negatively correlated with ppBMI in the -LNS group, but was not significant in the +LNS group. Argininosuccnic acid is an intermediate of the urea cycle that has been implicated in systemic nitric oxide production, but there has been no connection with argininosuccinate and obesity, insulin sensitivity, or general metabolic health in the literature to our knowledge. Most amino acids have been previously reported as being related to obesity and/or insulin resistance; we are likely underpowered to detect these differences in the present study.

BCAA concentrations were analyzed versus serologic markers of insulin resistance as well as all other amino acid concentrations in both –LNS and +LNS groups (**Supplemental Table 3**). Amino acids were grouped by essential versus non-essential status and Kyoto Encyclopedia and Genes and Genomes (KEGG) pathway map ID that included pathways related to BCAA Catabolism (map00280), Aromatic Amino Acid Metabolism (map00360), Alanine/Aspartate/Glutamate Metabolism (map00250), 1-Carbon/Methionine Metabolism (map00270), and the Urea Cycle/Arginine Metabolism (map00220). Correlations between BCAA and other amino acids in relation to LNS administration revealed loss of significance of several amino acids. These included Glutamate, Alanine, Glycine, Histidine, Ornithine, and Citrulline. With the exception of Histidine these are all non-essential amino acids, and should not be related directly to dietary intake. Positive correlations between BCAAs and Phenylalanine, Tyrosine, Aspartate, Methionine, and Serine (a mix of essential and non-essential amino acids) remained significant in mothers on and off LNS despite the fact that none were correlated with maternal ppBMI in mothers taking LNS.

All amino acids that correlated with maternal ppBMI including Tyrosine, Glutamate, Alanine, Methionine, Serine, Ornithine, and Citrulline, as well as Phenylalanine, Aspartate, Glycine, and Histidine were positively correlated with the BCAAs, as stated in the main text. Given the consistent correlations with maternal BMI in each of these amino acids, and the fact that those not correlated with maternal BMI are related by pathway to those that were, it is likely that the correlations between BCAA and these other amino acids are driven by maternal BMI. As discussed in the main text, while BCAAs are sentinel and established markers of metabolic health in obesity, the other amino acids and pathways, particularly Phenylalanine and Tyrosine, 1-Carbon metabolism, and Alanine/Aspartate/Glutamate have been similarly associated in metabolomics analyses.

**Table S1.** Nutrient Content of the Lipid-based Micronutrient Supplement (20g).

| Nutrient | Amount | Nutrient | Amount | Nutrient | Amount |
| --- | --- | --- | --- | --- | --- |
| Energy (kcal) | 118 | Iron (mg) | 20 | Thiamine (B1) (mg) | 2.8 |
| Protein (g) | 2.6 | Magnesium (mg) | 65 | Vitamin A (μg) | 800 |
| Fat (g) | 10 | Manganese (mg) | 2.6 | Vitamin B12 (μg) | 5.2 |
| Linoleic acid (g) | 4.59 | Niacin (mg) | 36 | Vitamin B6 (mg) | 3.8 |
| α-Linoleic acid (g) | 0.59 | Pantothenic acid (B5) (mg) | 7 | Vitamin C (mg) | 100 |
| Calcium (mg) | 280 | Phosphorous (mg) | 190 | Vitamin D2 (IU) | 1000 |
| Copper (mg) | 4 | Potassium (mg) | 200 | Vitamin E (mg) | 20 |
| Folate (μg) | 400 | Riboflavin (B2) (mg) | 2.8 | Vitamin K (μg) | 45 |
| Iodine (μg) | 50 | Selenium (μg) | 130 | Zinc (mg) | 15 |

**Table S2.** Regression analysis of leptin, adiponectin, insulin, and other amino acids in relation to maternal pre-pregnancy body mass index (ppBMI), taken at 12 weeks (12wks) gestation. “r-value (p-value)” are listed, and color coded (green = positive, red = negative) for direction of correlation if the p-value ≤ 0.05.

|  |  |  | **(-)LNS** | **(+)LNS** |
| --- | --- | --- | --- | --- |
| **Category** | **Pathway** | **Variable** | **vs ppBMI (r-value(p-value))** | **vs ppBMI (r-value(p-value))** |
| AA (Essential) | Phe&Tyr | **Phenylalanine** | 0.403 (0.078) | 0.152 (0.533) |
| AA (Non-Essential) | Phe&Tyr | **Tyrosine** | 0.481 (0.032) | 0.32 (0.181) |
| AA(Non-Essential) | Ala/Glu/Asp | **Glutamate** | 0.657 (0.002) | 0.257 (0.288) |
| AA(Non-Essential) | Ala/Glu/Asp | **Alanine** | 0.641 (0.002) | 0.138 (0.572) |
| AA(Non-Essential) | Ala/Glu/Asp | **Glutamine** | 0.276 (0.238) | -0.108 (0.66) |
| AA(Non-Essential) | Ala/Glu/Asp | **Aspartate** | 0.38 (0.098) | 0.06 (0.808) |
| AA(Essential) | 1-Carbon | **Methionine** | 0.47 (0.036) | -0.276 (0.253) |
| AA(Non-Essential) | 1-Carbon | **Glycine** | 0.346 (0.135) | -0.401 (0.088) |
| AA(Non-Essential) | 1-Carbon | **Serine** | 0.520 (0.019) | 0.109 (0.658) |
| AA (Essential) | His | **Histidine** | 0.383 (0.096) | -0.077 (0.754) |
| AA (Non-Essential, Non-Proteogenic) | Arg&Pro | **Ornithine** | 0.569 (0.009) | -0.045 (0.856) |
| AA (Non-Essential, Non-Proteogenic) | Arg&Pro | **Citrulline** | 0.521 (0.018) | -0.137 (0.576) |
| AA (Non-Essential) | Arg&Pro | **Arginine** | -0.003 (0.990) | 0.29 (0.229) |
| AA (Non-Essential, Non-Proteogenic) | Arg&Pro | **Argininosuccinic** | -0.587 (0.01) | -0.098 (0.707) |

**Table S3.** Correlations between branched chain amino acids ± LNS and serum parameters and other amino acids at 12 weeks gestation. “r-value (p-value)” are listed, and color coded (green = positive, red = negative) for direction of correlation if the p-value ≤ 0.05.
